# The Armadillo BTB Protein ABAP1 Is a Crucial Player in DNA Replication and Transcription of Nematode-Induced Galls

**DOI:** 10.3389/fpls.2021.636663

**Published:** 2021-04-30

**Authors:** Danila Cabral, Helkin Forero Ballesteros, Bruno Paes de Melo, Isabela Tristan Lourenço-Tessutti, Kércya Maria Simões de Siqueira, Luciana Obicci, Maria Fatima Grossi-de-Sa, Adriana S. Hemerly, Janice de Almeida Engler

**Affiliations:** ^1^INRAE, Université Côte d’Azur, CNRS, ISA, Sophia Antipolis, France; ^2^Instituto de Bioquímica Médica Leopoldo de Meis, Universidade Federal do Rio de Janeiro, Rio de Janeiro, Brazil; ^3^Departamento de Bioquímica e Biologia Molecular, Universidade Federal de Viçosa, Viçosa, Brazil; ^4^Laboratório de Interação Molecular Planta-Praga, Embrapa Recursos Genéticos e Biotecnologia, Brasília, Brazil

**Keywords:** ABAP1, *Arabidopsis thaliana*, cell cycle, galls, origin of replication, ROOT-KNOT nematode

## Abstract

The biogenesis of root-knot nematode (*Meloidogyne* spp.)-induced galls requires the hyperactivation of the cell cycle with controlled balance of mitotic and endocycle programs to keep its homeostasis. To better understand gall functioning and to develop new control strategies for this pest, it is essential to find out how the plant host cell cycle programs are responding and integrated during the nematode-induced gall formation. This work investigated the spatial localization of a number of gene transcripts involved in the pre-replication complex during DNA replication in galls and report their akin colocation with the cell cycle S-phase regulator Armadillo BTB Arabidopsis Protein 1 (ABAP1). ABAP1 is a negative regulator of pre-replication complex controlling DNA replication of genes involved in control of cell division and proliferation; therefore, its function has been investigated during gall ontogenesis. Functional analysis was performed upon *ABAP1* knockdown and overexpression in *Arabidopsis thaliana*. We detected *ABAP1* promoter activity and localized ABAP1 protein in galls during development, and its overexpression displayed significantly reduced gall sizes containing atypical giant cells. Profuse *ABAP1* expression also impaired gall induction and hindered nematode reproduction. Remarkably, *ABAP1* knockdown likewise negatively affected gall and nematode development, suggesting its involvement in the feeding site homeostasis. Microscopy analysis of cleared and nuclei-stained whole galls revealed that ABAP1 accumulation resulted in aberrant giant cells displaying interconnected nuclei filled with enlarged heterochromatic regions. Also, imbalanced *ABAP1* expression caused changes in expression patterns of genes involved in the cell division control as demonstrated by qRT-PCR. *CDT1a*, *CDT1b*, *CDKA;1*, and *CYCB1;1* mRNA levels were significantly increased in galls upon *ABAP1* overexpression, possibly contributing to the structural changes in galls during nematode infection. Overall, data obtained in galls reinforced the role of *ABAP1* controlling DNA replication and mitosis and, consequently, cell proliferation. *ABAP1* expression might likely take part of a highly ordered mechanism balancing of cell cycle control to prevent gall expansion. *ABAP1* expression might prevent galls to further expand, limiting excessive mitotic activity. Our data strongly suggest that *ABAP1* as a unique plant gene is an essential component for cell cycle regulation throughout gall development during nematode infection and is required for feeding site homeostasis.

## Introduction

Nematode-induced galls in plant roots are unusual tumor-like structures formed as a consequence of vascular tissue cell dedifferentiation and proliferation strongly engaging cell cycle reprogramming. The root-knot nematode (RKN) *Meloidogyne incognita* is an economically important species that must exploit and disturb the plant cell cycle to establish a successful parasitism (de Almeida-Engler et al., 1999). Several functional studies employing the model host *Arabidopsis thaliana* have shown that a number of cell cycle components are required for an effectual nematode infection (reviewed by de Almeida-Engler et al., 2011, 2015; Kyndt et al., 2016; Vieira and de Almeida Engler, 2017). Nematode feeding sites (NFS) are likely induced by nematode secretions and hold giant cells (GCs) used as the only nourishing source for the nematode (Vieira and Gleason, 2019). These giant-feeding cells are surrounded by neighboring cells (NCs) undergoing cycles of asymmetric cell divisions during initial gall genesis and require the activation and balance of mitotic and endocycle phases (de Almeida-Engler et al., 1999; de Almeida Engler and Gheysen, 2013; Vieira et al., 2013).

The eukaryotic cell cycle consists of four sequential phases (G1, S, G2, and M), when DNA is replicated at S phase and equally distributed into two daughter cells at mitosis (M phase) (Van’t Hof, 1985). The endocycle is an alternative cell cycle comprising recurrent S phases interceded by a G phase, resulting in an increase in cellular ploidy levels (Breuer et al., 2014). The passage across successive cell cycle phases is controlled by cyclin-dependent kinases (*CDKA;1* in Arabidopsis) that are activated upon binding to regulatory proteins such as mitotic cyclins, and by phosphorylation (Jeffrey et al., 1995; Nigg, 1995; Russo et al., 1996).

DNA replication is a cellular process regulated by the coordinated expression and action of numerous genes and proteins (Bell and Dutta, 2002; Shultz et al., 2007). During S phase, cells are licensed for DNA replication by the activation of the pre-replication complex (pre-RC) (Blow and Dutta, 2005; Brasil et al., 2017). The formation of the pre-RC starts with the sequential binding of ORC (Origin Recognition Complex) proteins to DNA replication origins, followed by the recruitment of additional DNA replication factors, like CDC6, CDT1a, CDT1b, and the minichromosome maintenance complex (MCM) (Bell and Dutta, 2002), culminating with the licensing of DNA for replication (Gutierrez et al., 2002; De Veylder et al., 2003; Machida and Dutta, 2004; Brasil et al., 2017). Plant cells exit the mitotic cycle and switch to the endocycle during differentiation, possibly using a similar DNA replication machinery, but the precise controls are still not clear. Cycles of DNA endoreduplication are likely essential for a number of plant cells to undergo differentiation routes and for proper organ development (Gutierrez, 2005), and this may include organ-like structures such as nematode-induced galls. Functional analyses of Arabidopsis mutant plants demonstrated that lack of pre-RC elements, like the *AtCDT1* homologs, *AtCDC6* and *AtORC2*, and two *AtMCM*s, may affect plant development by disturbing DNA replication, and consequently cell division, as well as the endocycle and heterochromatin structure (Springer et al., 2000; Holding and Springer, 2002; Castellano et al., 2004; Collinge et al., 2004; Dresselhaus et al., 2006).

The Armadillo BTB Arabidopsis Protein 1 (ABAP1) is a negative regulator of DNA replication and transcription that is specifically expressed in G1 and early S phase (Masuda et al., 2008). ABAP1 has been reported to be implicated in balancing cell division rates in leaves by negatively regulating pre-RC activity and DNA replication (Masuda et al., 2008). ABAP1 mechanism of action involves direct binding to members of the pre-RC and also association to transcription factors to negatively regulate the transcription of essential pre-RC genes (Masuda et al., 2008; Brasil et al., 2017). Plants overexpressing *ABAP1* present an overall decrease in cell numbers in leaves, and an opposite effect is observed in plants with downregulated levels of *ABAP1* (Masuda et al., 2008).

Thus, herein, we report for the first time the localization of a number of gall-expressed genes involved in DNA replication and report their akin colocation with the DNA replication inhibitor *ABAP1*. This study permitted us to decipher the function of a unique cell cycle inhibitor gene, *ABAP1*, in nematode-induced galls. Our tailored morphological and nuclear analysis shows that *ABAP1* knockdown resulted in increased mitotic activity in gall cells. Not surprisingly, overexpression of *ABAP1* inhibited gall development leading to less nematode reproduction. Cleared galls under markedly *ABAP1* expression confirmed decreased mitosis in galls brought by the excess of the inhibitory effect of the G1/S phase of the cell cycle. Finally, fluctuations upon down- and upregulation of *ABAP1* affected the expression of pre-RC and cell cycle control genes like *CDT1a*, *CDT1b*, *CYCB1;1*, and *CDKA;1. ABAP1* not only might regulate cell divisions in galls by repressing transcription of pre-RC genes or by associating to pre-RC complex apparatus but also might be remarkably affecting the endocycle. Our data further suggest that this unique ABAP1 protein might be an essential component of the cell cycle machinery controlling RKN-induced gall expansion, development and homeostasis.

## Materials and Methods

### Plant Material, Growth Conditions and Nematode Infection

Transgenic *ABAP1pro:GUS, ABAP1* over-expressor line (*ABAP1^*OE*^*), and a single T-DNA insertion mutant line with *ABAP1* knocked down expression (*ABAP1/abap1)* have been previously described by Masuda et al. (2008). *A. thaliana* Col-0 and genotype *Landsberg erecta* (LER) were used as the wild-type control of *ABAP1* knockdown (*ABAP1/abap1)* and *ABAP1* over-expressor line (*ABAP1^*OE*^*), respectively. Complete *ABAP1* knockout is lethal (Masuda et al., 2008); thus, we used single insertion heterozygote mutant lines (*ABAP1/abap1*). Seeds of Arabidopsis wild-type and transgenic lines were surface sterilized for 10 min in 5% NaOCl, followed by four washes with 95% ethanol and dried overnight. *ABAP1/abap1* line was selected in 50 mg/L kanamycin in germination medium. Plated seeds were kept in a growth chamber with 8-h light/16-h dark photoperiod at 21°C/18°C, respectively. After 4 weeks, Arabidopsis seedlings wild-type and healthy green plantlets of transgenic lines were placed in soil containing 30% of sand and were kept in a growth chamber in with 8-h light/16-h dark photoperiod at 21°C/18°C for growth for approximately 20 days. Each plant was then infected with 300 freshly hatched pre-parasitic stage 2 juvenile nematodes according to de Almeida-Engler et al. (2016).

### *In situ* Hybridization Analyses on Arabidopsis Galls

Galls undergoing high cell cycle activity (7–14 days after nematode inoculation-DAI) of Arabidopsis wild-type (Col-0) were dissected from infected seedlings, fixed in 2% glutaraldehyde, paraffin embedded, and sectioned to 10 μm with a microtome. All *in situ* hybridization stages have been executed essentially as explained by de Almeida Engler et al. (2009). Gene-specific sense and antisense radioactive probes of *CDC6, MCM5, ORC1, ORC2, ORC4, ORC5*, and *ORC6* were generated and hybridized with sectioned galls. *ORC1* AS probe will localize *ORC1a* and *ORC1b* homologs sharing 90% sequence similarity. Also, *CDC6* AS probe will localize *CDC6a* and *CDC6b* homologs. Microscopy slides filled with gall sections were developed, stained with 0.05% toluidine blue, and examined by dark-field optics. For transcript localization of *CDT1a* and *CDT1b* genes, all *in situ* hybridization steps have been executed essentially as explained by de Almeida Engler et al. (2009). Non-radioactive gene-specific sense and antisense probes were also generated for *CDT1a, CDT1b* localization. Hybridization procedure and probe synthesis for both *CDT1* probes was performed as described by de Almeida Engler et al. (2001).

### Microcopy Analysis of Promoter Activity and Protein Localization of *ABAP1*

Arabidopsis ABAP1 promoter activity was observed in uninfected roots (UR) and during nematode infection (3, 5, 7, 10, 14, and 21 DAI), and GUS activity was measured as previously described by de Almeida-Engler et al. (1999). GUS assays were performed overnight (12 h). Uninfected roots (UR) and galls of the *ABAP1pro:GUS* line (3, 5, and 10 DAI) were fixed in 2% glutaraldehyde and transferred to the chloral-lactophenol clearing solution (Beeckman and Engler, 1994). Remaining UR and gall samples were embedded in Technovit 7100 (Heraeus Kulzer) as described by the manufacturer and sectioned (3 μm) using standard microtomy. Whole mount and section images were taken with a digital Axiocam (Zeiss) with standard bright-field or dark-field optics.

### Immunocytochemical Assay

Galls 14 DAI of Arabidopsis wild-type cv. LER and *ABAP1^*OE*^* transgenic line were fixed in 4% formaldehyde in 50 mM Pipes buffer (pH 6.9). Galls and uninfected roots were dehydrated and embedded in butyl-methylmethacrylate essentially as described by Kronenberger et al. (1993) and Vieira et al. (2012). Primary anti-ABAP1 antibody and secondary antibody Alexa 488 goat anti-rabbit IgG (Molecular Probes, Eugene, OR, United States) were diluted 100- and 300-fold, respectively, in blocking solution (1% bovine serum albumin in 50 mM Pipes buffer, pH 6.9, and 0.2% DMSO). As control, primary antibody was omitted in some slides. Samples were then washed twice 15 min in Pipes buffer. A 2-h incubation at 37°C was performed with the secondary antibody. DNA was stained with 1 μg ml^–1^ 4′,6-diamidino-2-phenylindole (Sigma-Aldrich) in water, briefly rinsed, and mounted in glycerol 90%. Samples were observed under a microscope (Axioskop, Zeiss, Jena, Germany) equipped for epifluorescence microscopy, and images were acquired with a digital camera (Axiocam, Zeiss).

### Histological Analysis and Nematode Infection Assays

For nematode infection tests and histological analysis, plantlets from Arabidopsis wild-type (cv. Col-0 and LER) and transgenic lines (*ABAP1/abap1* and *ABAP1^*OE*^*) germinated in soil were infected with 300 juvenile nematodes (J2) of *M. incognita* per plant (de Almeida-Engler et al., 2016). Plantlets were then kept in a growth chamber (8-h light/16-h darkness) during 6–7 weeks after nematode inoculation. The numbers of galls and egg masses for the two biological repetitions of transgenic lines were recorded and compared to wild-type plants. For morphological analyses of each line, galls were dissected at different time points after inoculation (3, 7, 14, and 21 DAI), were fixed in 2% glutaraldehyde in 50 mM Pipes buffer, pH 6,9, and then dehydrated and embedded in Technovit 7100 (Heraeus Kulzer) as depicted by the manufacturer. Embedded roots and galls were sectioned (3 μm), stained in 0.05% toluidine blue, and mounted in DPX (Sigma-Aldrich). Microscopic examinations were performed under bright-field optics and images were taken with a digital camera (Axiocam, Zeiss). In addition, subsequent gall tissue sections used for histological analysis were stained with 1 μg/ml 4,6-Diamidino-2-phenylindole (DAPI) and mounted in 90% glycerol on a microscope slide and cover-slipped. Nuclear staining was then observed by fluorescence microscopy (Axioplan 2, Zeiss) equipped for epifluorescence.

### Measurements of GC Area and Gall Diameter of *ABAP1* Transgenic Lines

Two of the largest GCs per gall section (21 DAI) of the *ABAP1/abap1* and *ABAP1^*OE*^* at different time points after nematode infection (7, 14, 21, and 40 DAI) were surface measured using the Axioplan 2 (Zeiss) software. A minimum of 30 GCs and diameter of galls per transgenic line were measured at each time point. Measurements were analyzed by two-way analysis of variance (ANOVA), and significant differences between average values were identified by Tukey–Kramer test using PROC GLM in SAS (version 9.1.3, SAS Institute Inc., Cary, North Carolina).

### Nuclei Morphology Analysis of *ABAP1* Lines

*ABAP1/abap1*, *ABAP1^*OE*^*, and wild-type mature galls (21 DAI) were dissected, mounted in 3% agarose (Thermo-Fischer, United States), and sectioned with a vibratome (Microm HM650V) to 80–100 μm. Nuclei of cleared galls were DAPI stained as described by Antonino de Souza Junior et al. (2017) and analyzed by confocal microscopy (Zeiss LSM 880). Samples were mounted in 90% glycerol between two coverslips in order to be able to observe both sides. Stacks were generated from approximately 50 images per sample with approximately 1 μm optical slice thickness. Dye excitation was done with a diode 405-nm laser and fluorescence was collected between 431 and 532 nm.

### Ploidy Level Analyses of *ABAP1* Transgenic Lines

Flow cytometry analysis was performed on uninfected roots and galls (21 DAI) of *ABAP1/abap1, ABAP1^*OE*^*, and wild-type (cv. Col-0 and LER). Samples were chopped for 2 min with a razor blade in a buffer solution containing 300 μl of 45 mM MgCl_2_, 30 mM sodium citrate, 20 mM MOPS, pH 7.0, and 0.1% Triton X-100, as previously described by Vieira et al. (2013). Harvested plant material was filtered in a 70-μl mesh and stained in 1 μg/ml of DAPI. The previous steps were performed twice. The ploidy levels were measured with the LSRII Fortessa (BD Biosciences) flow cytometer and the BD FACS Diva software (BD Biosciences). An average of 100,000 nuclei per run was collected for uninfected roots, the fraction of nuclei with ploidy levels from 2C to 16C was expressed as a percentage of the total number of nuclei measured. For gall tissues, a minimum of 30 galls (40 DAI) were extracted and pooled for each independent trial, composed of two technical and two biological repetitions for each line. For galls, ±30,000–100,000 events/run nuclei per run were collected. The mean values of repetitions of independent trials were calculated, and the fraction of nuclei at ploidy levels from 2C to 64C was expressed as a percentage of the total number of nuclei recorded.

### Acid Fuchsin Staining of *ABAP1* Transgenic Lines Infected Roots

Acid fuchsin staining was performed in infected roots of *ABAP1/abap1*, *ABAP1^*OE*^*, and wild-type seedlings as described by de Almeida-Engler et al. (2016), to track nematode development within the roots. The whole infected roots (40 DAI) were fixed and stained for 5 h in a solution of equal parts of 95% ethanol and glacial acetic acid, containing 17.5 mg/L acid fuchsin. The root tissue and galls were distained by incubating in a solution of chloral hydrate (0.2 g/ml in water) for 16 h. After rinsing the roots several times with tap water, roots containing nematodes were stored in acidified glycerol (five drops of 1.0 M HCl were added to 50 ml of glycerol). Nematode-infected roots were observed with a digital camera (Axiocam; Zeiss).

### Quantitative Real-Time PCR (qRT-PCR)

The expression profile of genes involved in DNA replication and cell cycle progression, as well as genes directly regulated by ABAP1, was investigated by qRT-PCR. Total RNA was extracted from isolated galls 14 DAI, using Trizol reagent (Thermo Fisher Scientific) according to the manufacturer’s guidelines. The RNA was then treated with RNase-free DNase I (Thermo Fisher Scientific) before reverse transcription. One microgram of treated RNA was used to synthetize the cDNA using a SuperScript III Reverse Transcriptase (Thermo Fisher Scientific) synthesis kit using oligo DT primers. This cDNA (1/20) was then amplified using SYBR Green I PCR Master No Rox (Eurogentec, Angers, France), and amplification of *PCNA1, CDT1a, CDT1b, CYCB1;1, CDKA;1, SOG1, E2Fa*, and *TCP24* genes was performed with each specific primer pair (Supplementary Table 1). Relative expression was calculated using the 2-ΔΔCT method with Oxaloacetate and NADPH as constitutive genes. qRT-PCR values are means from three biological replicates.

## Results

### Transcripts of Representative Pre-RC Genes Are Expressed in Galls

**FIGURE 1:**
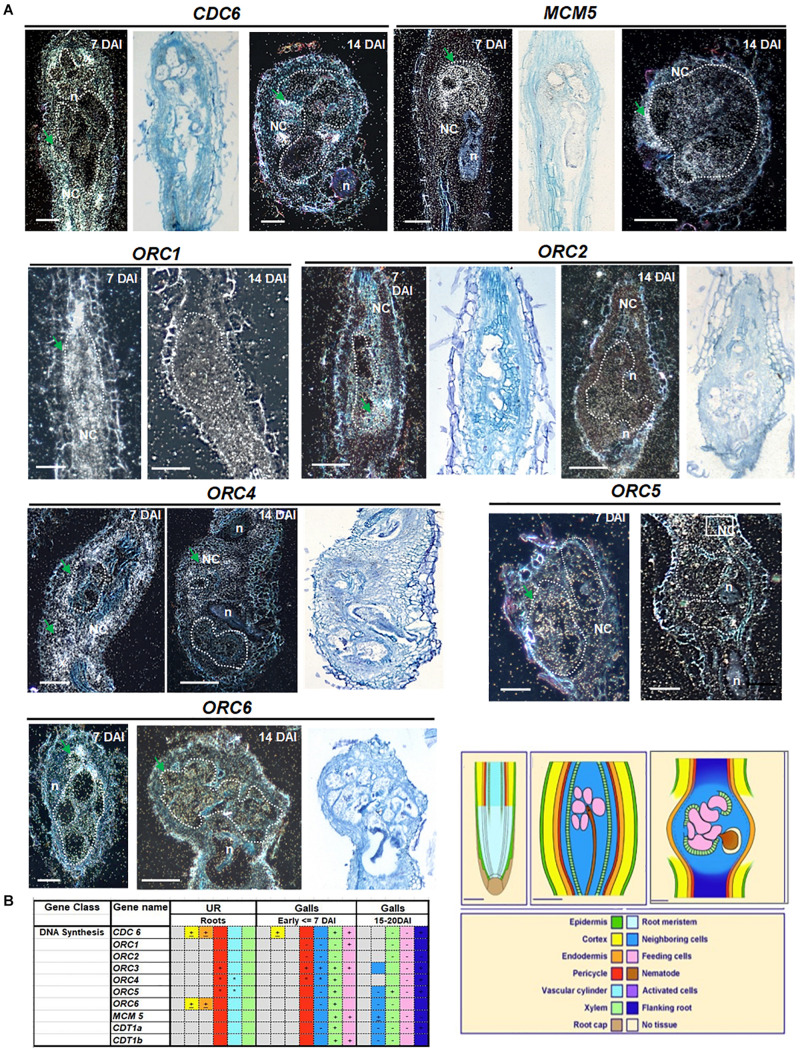
Cell cycle pre-replication complex members: *CDC6, MCM5, ORC1, ORC2, ORC3, ORC4, ORC5*, and *ORC6* localization in *Meloidogyne incognita*-induced galls of Arabidopsis. mRNA *in situ* localization illustrating transcripts of *CDC6, MCM5, ORC1, ORC2, ORC3, ORC4, ORC5*, and *ORC6* in *Meloidogyne incognita*-induced galls in Arabidopsis roots and summarized data in tissue color table. **(A)** Dark- and bright-field images of *CDC6, MCM5, ORC1, ORC2, ORC4, ORC5*, and *ORC6* showing mRNA *in situ* localization in 7 and 14 days after inoculation galls. Sections were hybridized with 35S-labeled antisense (AS) RNA probes and the hybridization signal is shown as white dots under dark-field optics and black dots under bright-field optics. Regions with high silver grain concentrations are depicted with green arrows. Feeding cells are surrounded by dotted lines. **(B)** Table summarizing transcript localization and hybridization intensities of genes analyzed involved in DNA synthesis. Colors in the table and drawings illustrate different tissues of an uninfected root and of young (<7 DAI) and mature galls (15–21 DAI). Patterns described in the table are based on at least 30 sections of each time point. Signs within colored squares represent intensity of signal: +, strong; -, weak; ±, sometimes expressed and *, patchy expression, based on visual recording of silver grain concentration. Colored squares with no sign mean intermediate levels of expression. Gray colors mean no signal, often observed for epidermis and cortical tissues. DAI, days after inoculation; n, nematode; NC, neighboring cells; UR, uninfected root. Scale bars drawings and images = 50 μm.

To investigate the replication licensing system in galls, we performed *in situ* hybridization with a number of partners of the pre-RC involved in DNA replication during different phases of feeding site development. At 7 DAI, galls are ongoing intense mitotic activity, whereas at 14 DAI, they entered the endocycle phase. All genes analyzed were expressed in gall cells at varying intensities (*CDC6, MCM5, ORC1, ORC2, ORC3, ORC4, ORC5, ORC6, CDT1a*, and *CDT1b*) (Figure 1 and Supplementary Figure 1). Illustrated images of *MCM5*, *ORC1, ORC4*, and *ORC5* shows apparently stronger hybridization signal at 7 DAI, whereas *ORC2* and *ORC6* showed similar intensities at 7 and 14 DAI in feeding cells. *CDC6* and *ORC4* expression was high in dividing NCs (Figure 1). Only *ORC6* showed a quite strong hybridization signal at 14 DAI. Overall results are illustrated in the table in Figure 1B, which may slightly differ from images since it reflects observations performed on at least 50 galls. Both *CDT1a* and *CDT1b* were later analyzed by non-radioactive *in situ* hybridization and revealed that both genes were expressed in uninfected root vascular tissue and in galls 7 and 14 DAI. For all experiments, control hybridizations resulted in no signal. Results of hybridizations in uninfected roots have been previously reported and illustrated in de Almeida Engler et al. (2012).

### ABAP1 Is Expressed in Root-Knot Nematode-Induced Galls

**FIGURE 2:**
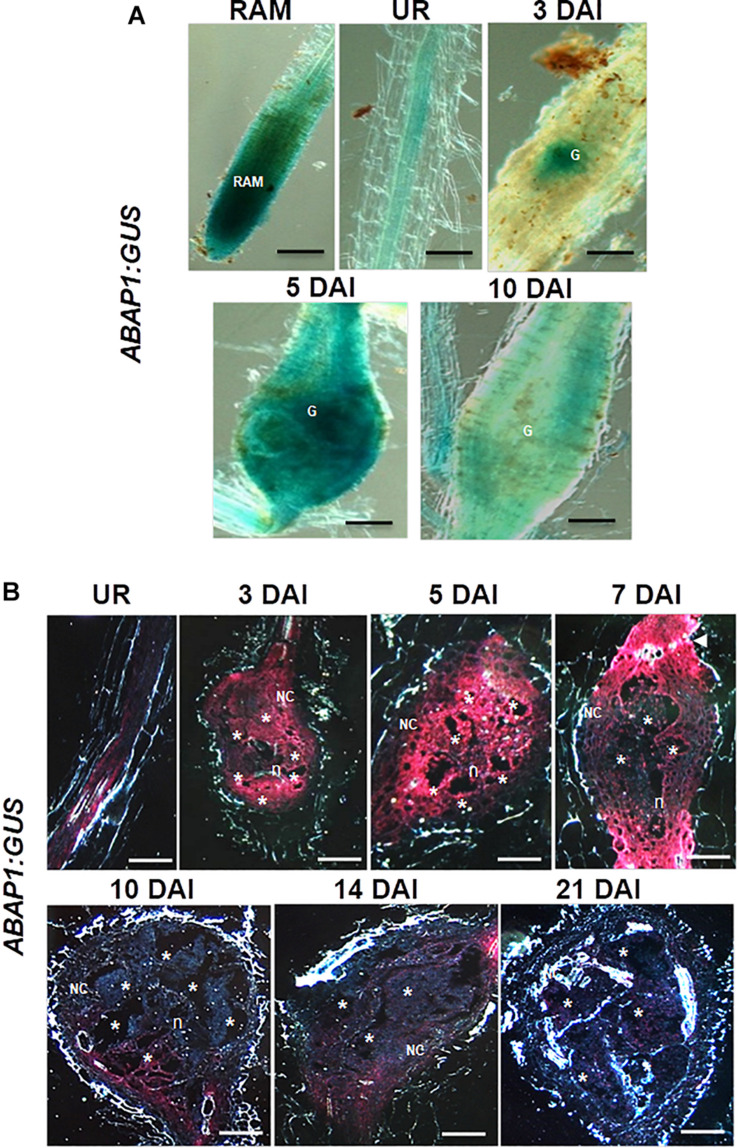
Expression pattern of the *Arabidopsis ABAP1pro:GUS* line in uninfected roots and in *M. incognita*-induced galls. **(A,B)** Illustrate *ABAP1* promoter activity in the root apical meristem (RAM), in an uninfected root (UR) and during gall development (3, 5, 7, 10, 14, and 21 days after inoculation). Bright-field images of whole roots and galls illustrate the GUS staining in blue **(A)** and dark-field images show GUS staining in red **(B)**. G, gall; Asterisk, giant cell; NC, neighboring cells; n, nematode. Scale bars = 50 μm.

The promoter activity of *ABAP1* was examined by carrying out GUS assays in uninfected roots and in Arabidopsis galls at different stages of development (3, 5, 7, 10, 14, and 21 DAI). *ABAP1* was expressed in the root apical meristem (RAM) and weakly in vascular root tissue of uninfected roots (Figure 2A). GUS staining performed on entire galls illustrated an early (3 DAI) and localized induction of *ABAP1* promoter activity, which then spread along all gall cells (5 DAI), correlating with the increased cell cycle activity in galls. Sectioned galls revealed intense GUS staining in GCs and in NCs at 3 and 5 DAI decreasing at 7 DAI as gall matured (Figures 2A,B). Expression was then significantly decreased at 10–14 DAI and nearly disappeared at later stages of gall expansion (21 DAI). It was notable that at 10 and 14 DAI, GUS staining was stronger in NCs, which are still proliferating close to the endoreduplicating GCs. The lowered but still present promoter activity in GCs at 7–14 DAI suggests the involvement of ABAP1 expression in the GC endocycle phase. In addition, lowered *ABAP1* expression in NCs after 7 DAI coincides with the decreased mitotic activity in galls at later stages. Interestingly, GUS staining remained strong only in root zones flanking the gall (Figure 2B illustrated in gall at 7 DAI). This observation suggests that *ABAP1* expression might be somehow linked to the presence of a neighboring gall.

**FIGURE 3:**
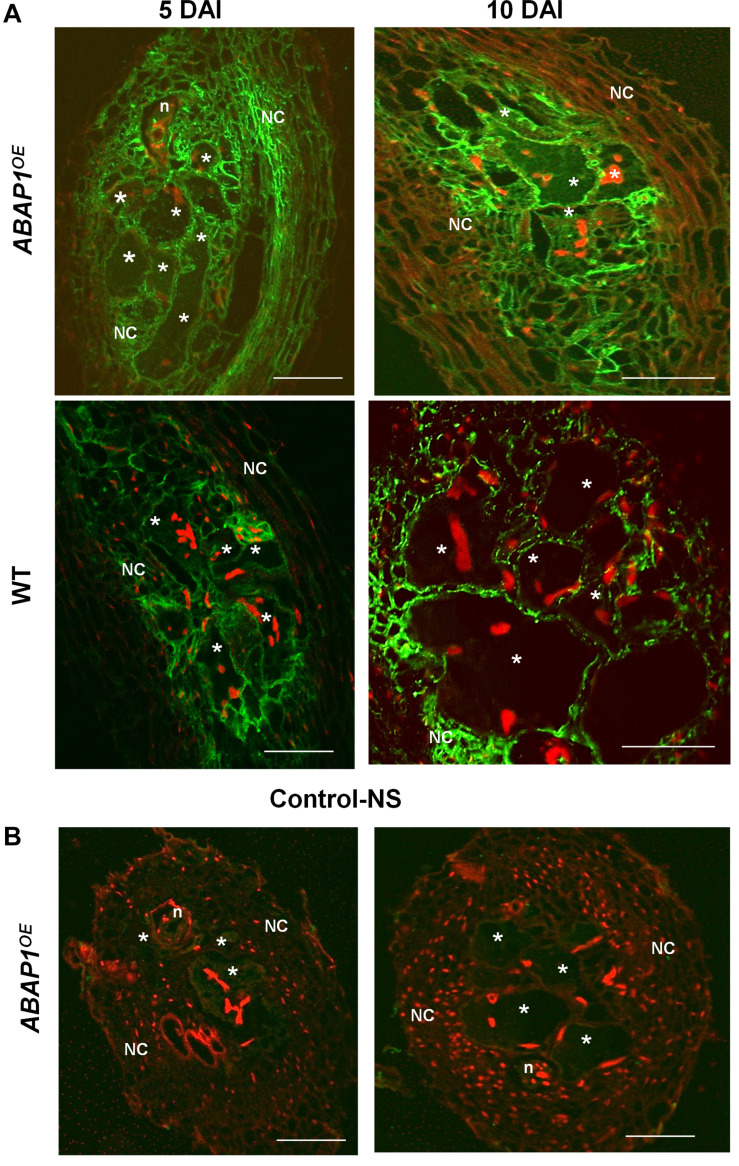
ABAP1 localization in *M. incognita*-induced galls. **(A)** Immunofluorescence of ABAP1 (green) in wild-type and ABAP1^*OE*^ galls 5 DAI and 10 DAI. **(B)** Images show galls 5 and 10 DAI treated with the normal serum (NS). DAPI-stained nuclei are visualized in red. Asterisk, giant cell; n, nematode, NC, neighboring cells. Scale bars = 50 μm.

Immunocytochemical analysis of ABAP1 protein confirmed promoter GUS analysis in galls and revealed a visible fluorescence localized in GCs and NCs within galls during the mitotic stage (5 DAI) decreasing but still present during the endocycle stage (10 DAI; Figure 3A). No clear fluorescence was detected in the differentiated cortex or epidermis tissues bordering the galls, which normally do not undergo mitotic activity since later these cells will flop off (Figure 3A). Strongest fluorescence was detected in *ABAP1*^*OE*^ galls and a weaker pattern was seen in wild-type galls. No fluorescence was detected in *ABAP1^*OE*^* galls treated with the normal serum used as a control (Figure 3B).

### *ABAP1* Knockdown Expression in Arabidopsis Reduced the Gall Size and Affected Nematode Development

**FIGURE 4:**
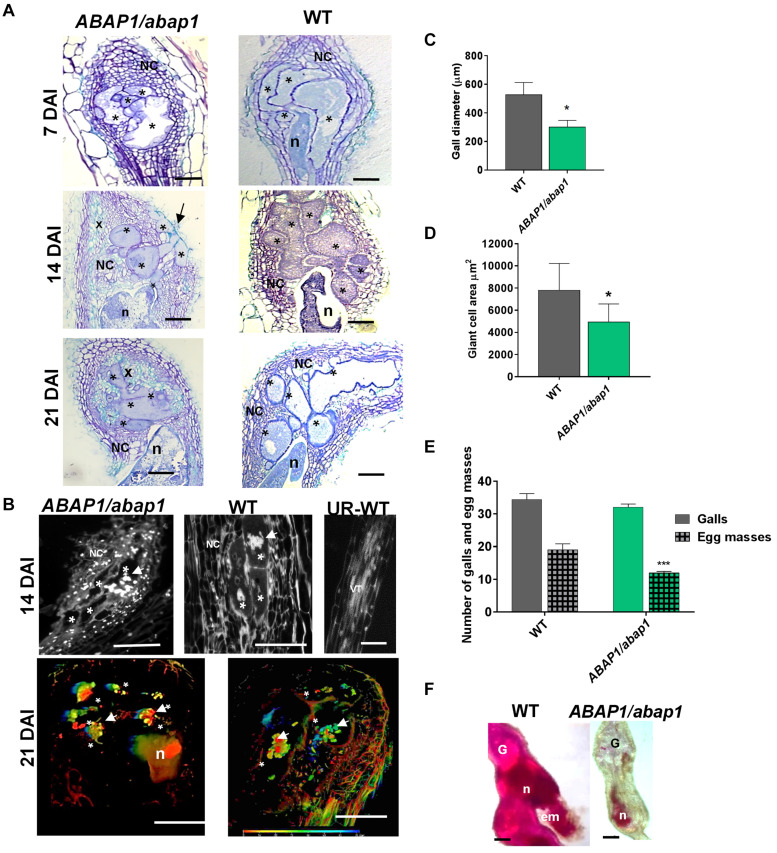
Histological analysis and nuclei morphology of galls, and resistance tests of *ABAP1* knockdown plants infected with *M. incognita*. **(A)** Bright-field images of gall sections of *ABAP1/abap1* and wild-type (7, 14, and 21 DAI) stained with toluidine blue. Note abnormally convoluted giant cells extended to the gall border (arrow 14 DAI) and xylem (x) proliferation (14 and 21 DAI) upon *ABAP1* knockdown. **(B)** Gall 21 DAI and uninfected root sections of *ABAP1/abap1* and wild-type stained with 4,6-diamidino-2-phenylindole (DAPI); and 3D confocal projections of serial optical sections of wild-type (WT) and *ABAP1/abap1* galls 21 DAI nuclei (white arrows), cleared and DAPI stained. Colored bars represent the depth of nuclei within the gall. DAI, days after inoculation; asterisk, giant cell. Bars = 50 μm. **(C)** Gall diameter measurements revealed that *ABAP1/abap1* plants have smaller galls than wild-type. **(D)** Giant cell measurements showed smaller giant cell area in the *ABAP1* knockdown line compared to the wild-type. **(E)** Resistance test of *ABAP1/abap1* line showed a significant reduction of eggs mass number. **(F)** Galls (40 DAI) from these resistance tests were acid fuchsin stained and showed a delay in nematode development under low *ABAP1* levels. Statistical analysis was performed *P < 0.05) or ***P < 0.01 based on Student’s *t* -est. Asterisk, giant cell; NC, neighboring cells; n, nematode; x, xylem; G, gall; em, egg mass. Scale bars = 50 μm.

To address whether *ABAP1* downregulation could disturb gall induction as well as nematode development, we used a single insertion mutant line (*ABAP1/abap1*), considering that full *ABAP1* knockout is lethal (Masuda et al., 2008). A detailed morphological analysis of *ABAP1/abap1* galls illustrated small vacuolated GCs with cell wall stubs during the mitotic phase (7 DAI) and increased NCs proliferation compared to wild-type galls (Figure 4A). As galls developed, these remained smaller than wild-type even when the visible increased density of xylem elements was remarked (Figure 4A). Gall measurements in infected *ABAP1/abap1* line confirmed their reduced sizes in diameter as well as decreased GC area compared to wild-type (Figures 4C,D). Thus, even when a plethora of NCs and xylem divisions are visible, galls are still apparently smaller than in wild-type. To examine whether *ABAP1* knockdown had an influence on nematode development and reproductive ability, infection tests were performed. Although no significant changes in gall numbers was observed among *ABAP1/abap1* and wild-type plants, a significant reduction in egg mass production was observed (Figure 4E). Also, nematode development in *ABAP1/abap1* plants showed a clear delay or arrest in maturation (Figure 4F). DAPI-stained gall sections 14 DAI in *ABAP1/abap1* revealed small grouped nuclei and extra NCs compared to a wild-type gall (Figure 4B and Supplementary Movies 1, 2). 3D reconstruction of *ABAP1/abap1* galls cleared and DAPI stained revealed that nuclei were small, clumped, and more circuitous compared to the wild-type and GCs were apparently smaller than wild-type (Figure 4B, Supplementary Movies 1, 2).

### *ABAP1* Overexpression Had a Noteworthy Negative Impact in Gall and Nematode Development

**FIGURE 5:**
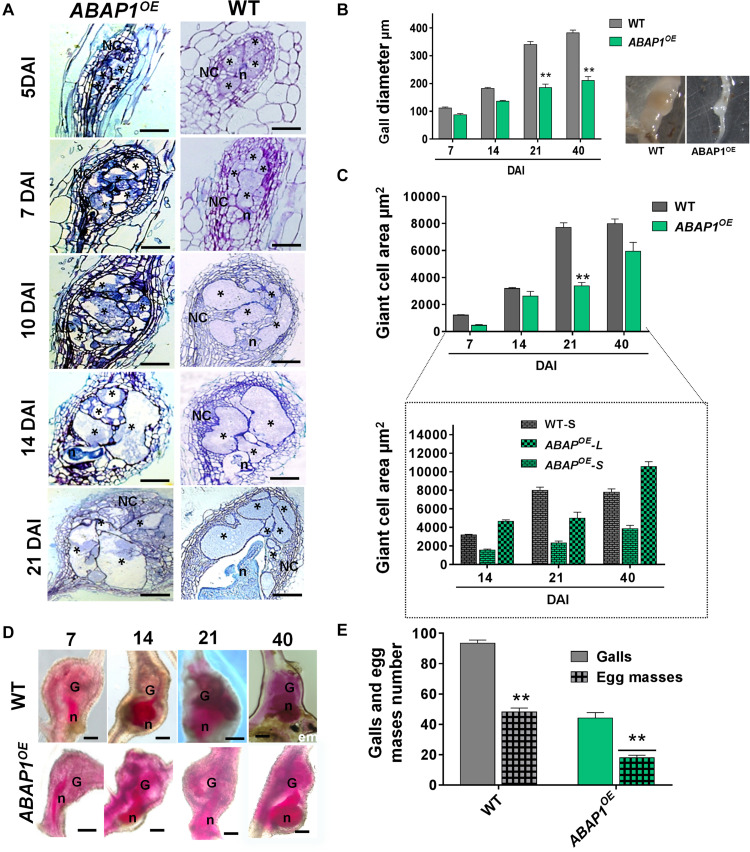
Ectopic *ABAP1* expression affects gall morphology and revealed feeding sites with reduced sizes leading to decreased nematode reproduction. **(A)** Histological analysis of galls overexpressing *ABAP1^*OE*^* compared to wild-type (WT) at different stages of nematode infection (5, 7, 10, 14, and 21 DAI). Bright-field micrographs show longitudinal gall sections stained with toluidine blue. **(B,C)** Gall diameter and giant cell area of *ABAP1^*OE*^* galls compared with WT. Note that galls from *ABAP1^*OE*^* are smaller than WT galls and two sizes categories of giant cells in *ABAP1^*OE*^* were observed: small and cytoplasm filled giant cells (*ABAP1^*OE*^-S*) and large containing large vacuoles (*ABAP1^*OE*^-L*). **(D)** Acid fuchsin staining of *ABAP1^*OE*^* and WT galls at different developmental stages (7, 14, 21, 40 DAI) of nematode development. A visible delay or arrest in nematode development is seen in the *ABAP1^*OE*^* line consequently inhibiting and delaying egg mass laying. **(E)** Nematode infection tests of *ABAP1^*OE*^* showed a significant decrease in galls and egg masses numbers compared to the WT. Data shown represent means ± SD from two experiments with *n* = 20 plants per line. Statistical differences are marked with *P < 0.05) or **P < 0.01 based on Student’s *t*-test analysis. Asterisk, giant cell; NC, neighboring cells; n, nematode; G, gall; em, egg mass. Scale bars = 50 μm.

**FIGURE 6:**
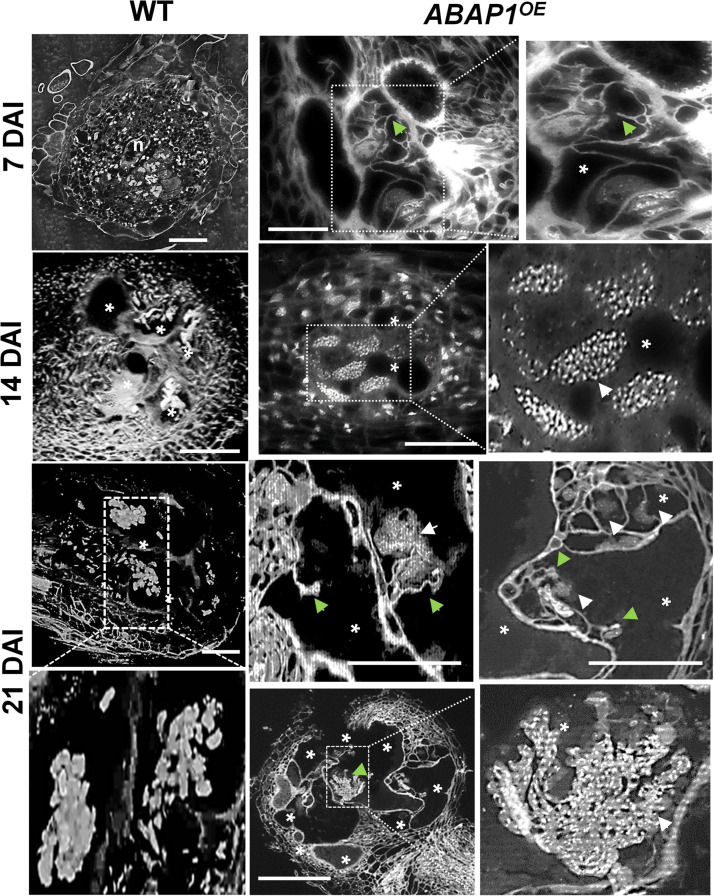
*ABAP1* overexpression induces nuclear morphology changes in root-knot nematode-induced giant cells. Gall nuclei from *ABAP1^*OE*^* and wild-type (WT) plants, stained with 4,6-Diamidino-2-phenylindole (DAPI), were observed under dark-field optics. Numerous large stained nuclear dots (white arrows) likely illustrate condensed chromatin at different stages of gall development (7, 14, and 21 DAI). Abnormal cell wall stubs (green arrows) were also seen in *ABAP1^*OE*^* galls. Strong cellular and nuclei phenotypes in mature galls suggest cumulative mitotic defects upon ABAP1 overexpression. DAI, days after inoculation; asterisk, giant cell; n, nematode. Scale bars = 50μm.

The effects on cell proliferation and tissue defects of increased *ABAP1* concentration were obvious during gall ontogeny (5, 7, 10, 14, 21 DAI) as visualized by histological analysis. Seen in longitudinal gall sections, GCs presented irregular forms, were more vacuolated, and illustrated severely reduced division of NCs (Figure 5A). Cell wall stubs present in GCs suggested additional mitotic defects than normally observed in wild-type galls. Reduced gall diameter measurements confirmed decreased mitotic activity in ABAP1^*OE*^ galls. Statistical decrease in gall size was seen mainly during expansion around 21 DAI and remained visibly small until gall fully matured (40 DAI) (Figure 5B). The average of GC area in the *ABAP1^*OE*^* (after 21 DAI) was also statistically significantly smaller than in wild-type (Figure 5C). No statistical differences of GCs of mature galls (40 DAI) were due to the regularly observed two populations of GC sizes within the same galls, named small (*ABAP1^*OE*^-S)* and large (*ABAP1^*OE*^-L)* (Figure 5C). Two types of GC morphologies were observed in a same feeding site: GCs with smaller areas containing a dense cytoplasm and others containing large vacuoles with increased area (Figures 5A, 6). Acid fuchsin stain allowed us to remark the delayed nematode development in *ABAP1^*OE*^* galls in contrast to wild-type infected roots at 7, 14, 21, and 40 DAI (Figure 5D). A large fraction of nematodes remained as parasitic second-stage juveniles and were associated with reduced size feeding sites within these transgenic roots (Figure 5D). Infection tests revealed an approximately 50% reduction in gall number in the *ABAP1^*OE*^* line, followed by a statistically significant reduction in egg mass number compared with infected wild-type control roots (Figure 5E).

### Increased *ABAP1* Expression Led to Aberrant Nuclear Pattern in GCs

**FIGURE 7:**
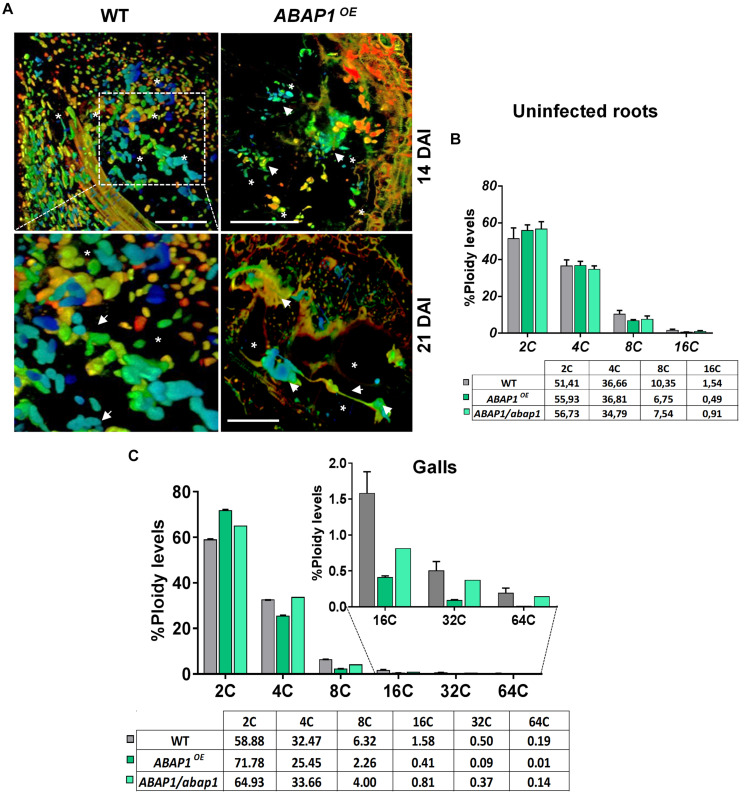
*ABAP1* overexpression affects nuclei morphology and ploidy levels in *M. incognita-induced* galls. **(A)** 3D confocal projections of serial optical sections of wild-type (WT) and *ABAP1^*OE*^* galls 14 and 21 DAI nuclei (white arrows), cleared and stained with 4,6-diamidino-2-phenylindole (DAPI). Elongated and apparently connected nuclei are observed in giant cells in *ABAP1^*OE*^* galls 21 DAI. DAI, days after inoculation; asterisk, giant cell. Bars = 50 μm. **(B,C)** Flow cytometry analysis and ploidy levels (percentage) of uninfected roots **(B)**, and galls of *ABAP1^*OE*^* and *ABAP1/abap1* lines compared to WT **(C)**. The ploidy levels were kept high for GCs in *ABAP1/abap1* as in WT and decreased progressively from 8C to 64C in *ABAP1^*OE*^*, when compared to *ABAP1/abap1* and WT galls. For each line and experiment, three independent biological repetitions (± 30 galls) were performed. Scale bars = 50μm.

The effect of surplus *ABAP1* expression in the nuclear structure and to a certain level gall morphology was observed in detail by DAPI-stained semi-thin sections and in thick slices by confocal microscopy (Figures 6, 7A and Supplementary Movie 4). DAPI-stained gall slices 7, 14, and 21 DAI revealed GCs with variable sizes quite vacuolated and the frequent presence of cell wall stubs, flanked by nuclei in enlarged GCs suggestive of aberrant mitotic activity. Remarkably, nuclei were clustered, apparently displaying irregular shapes, and presented a large number of bright fluorescing condensed chromatin dots. We then performed 3D nuclei reconstruction generating thick slices of *ABAP1^*OE*^* galls cleared and DAPI stained to better picture nuclear defects that undeniably became more pronounced as galls matured (Figure 7A and Supplementary Movie 4). Interconnected nuclei were correlated with the presence of the *ABAP1* overexpression, and this phenotype was not present in wild-type GC nuclei (Figure 7A, Supplementary Movies 3, 4). Nuclear ploidy levels in uninfected roots did not show significant differences among *ABAP1/abap1*, *ABAP1^*OE*^*, and wild-type (Figure 7B). The decreased 4C DNA ploidy levels in *ABAP1^*OE*^* galls most probably derived from the hindered NC division (Figure 7C). Comparable lessened 8C–64C levels in *ABAP1^*OE*^* galls, challenged to *ABAP1/abap1* and control wild-type (Figure 7C), might be derived from the inhibited mitotic activity ensuing in aberrant apparently connected nuclear phenotypes.

### Increased or Decreased Levels of *ABAP1* Affect the Regulation of Cell Cycle Genes Expression in Galls

**FIGURE 8:**
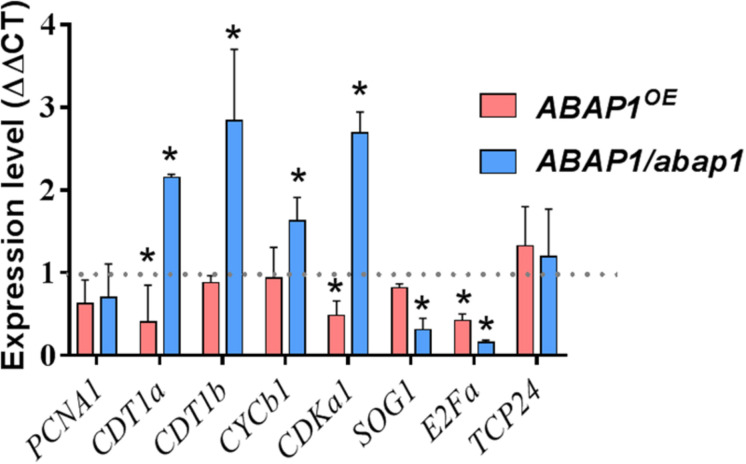
qRT-PCR assays revealed a regulation of cell cycle genes in galls induced by *M. incognita* in *ABAP1^*OE*^* and *ABAP1/abap1* plants. *PCNA*, *CDT1a*, *CDT1b*, *CYCB1;1*, *CDKA;1, SOG1*, and *E2Fa* were up- or downregulated upon knockdown or overexpression of *ABAP1* in galls. The analysis was performed in galls 14 days after inoculation, when cell cycle activity is high. *AtOxaloacetate* and *AtNAPDH* genes were used as endogenous reference genes. Data represent mean ± SEM of three independent experiments. The values were normalized according to mRNA levels of wild-type controls (WT-Col-0 for *ABAP1/abap1* and WT-Ler for *ABAP1^*OE*^*) (dashed black line). Asterisks indicate values statistically different from the wild-type (Student’s *t*-test, *P* = 0.05).

**FIGURE 9:**
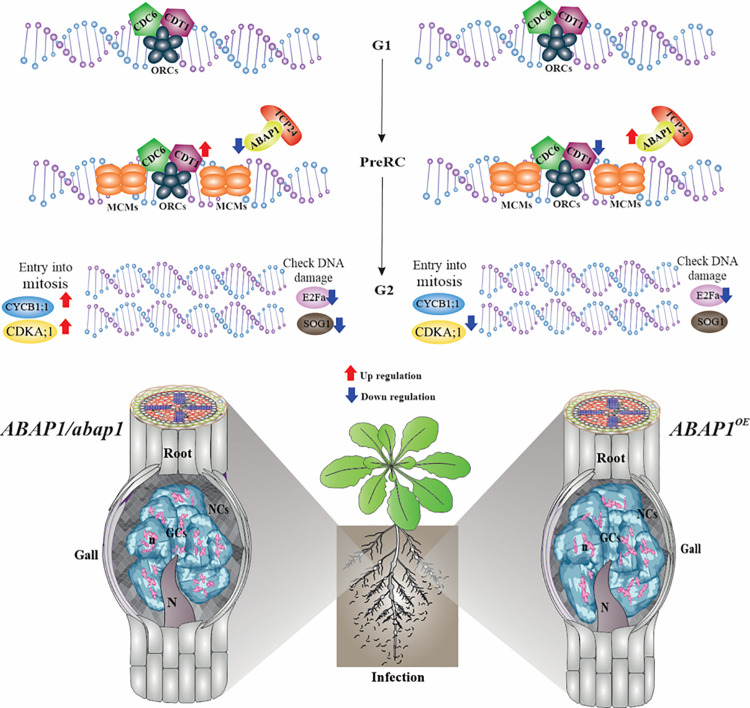
Schematic view and proposed model for *ABAP1* control in galls during DNA replication and S-phase progression. *ABAP1* expression senses and responds to intra- and extracellular signals like nematode infection, leading to changes in the expression pattern of gene members of the pre-replication complex and other S-phase genes. Genes analyzed by *in situ* hybridization are illustrated on the upper DNA drawing, and genes analyzed by qRT-PCR are shown with arrows of up- and downregulation upon increased (red arrows) or decreased (blue arrows) *ABAP1* expression. When *ABAP1* is downregulated (left side), genes like *CDT1*, *CDKA;1*, and *CYCB1;1* increased their expression levels and galls showed more mitotic activity also illustrated in the drawing on the left side. On the other hand, when the *ABAP1* is overexpressed (right side), decreased activity of the pre-RC can possibly regulate negativity of other partakers of the cell cycle such as *CDT1* and CDK1 decreasing mitotic events and inhibiting DNA replication in galls. These might lead to aberrant forms of the nuclei in giant cells illustrated at the right side. Overall, these changes might drastically affect the nematode development and reproduction.

To extend our knowledge of *ABAP1* function during gall development, mRNA levels of genes involved in DNA replication and cell cycle progression, as well as genes directly regulated by ABAP1, were investigated by qRT-PCR. The following described genes were chosen to be investigated in galls 14 DAI in *ABAP1^*OE*^*, *ABAP1/abap1*, and wild-type plants. The TCP24, a class-II TCP transcription factor family, negatively regulates plant cell proliferation and leaf morphogenesis when associated with ABAP1 (Cubas et al., 1999; Nath et al., 2003; Palatnik et al., 2003). *PCNA1* (Proliferating Cell Nuclear Antigen) is an auxiliary protein for DNA polymerase that is highly expressed during the S phase of the cell cycle and is widely used as an index of the proliferative cell activity in cancer tissues and plant cells (Strzalka and Ziemienowicz, 2011; Yokoyama et al., 2016). *CDT1* (Chromatin Licensing and DNA Replication Factor 1) is required for both DNA replication and chromosome segregation, and in mammalian cells, small changes in *CDT1* control can lead to catastrophic consequences for genome stability (Brasil et al., 2017). Moreover, *CDT1a* and *CDT1b* are direct targets of ABAP1 transcription repression in Arabidopsis, being used as a marker of ABAP1 levels and activity (Masuda et al., 2008; Brasil et al., 2017). *CYCB1;1* (Cyclin-B1-1) and *CDKA;1* (Cyclin-dependent kinase A-1) are core proteins driving cell cycle control (De Veylder et al., 1997, 2003; Coudreuse and Nurse, 2010). The transcription factor E2Fa is implicated in cellular proliferation and endocycle stimulation (De Veylder et al., 2002; Magyar et al., 2012). Ultimately, *SOG1* (suppressor of gamma-response 1) is a cell cycle checkpoint control transcription factor that functions downstream of the ATR and ATM pathway and is required for both cell cycle arrest and the induction of DNA repair genes (Yoshiyama et al., 2009). In our qRT-PCR analyses, no significant difference was seen on the mRNA levels of *TCP24* and *PCNA1* among *ABAP1^*OE*^*, *ABAP1/abap1*, and wild-type galls, suggesting that minimal concentrations might be enough for replication progression. However, *CDKA;1* expression was significantly repressed in *ABAP1^*OE*^* galls, and *CDKA;1* and *CYCB1;1* were induced in *ABAP1/abap1* galls compared to wild-type (Figures 8, 9). These data corroborate with a negative regulation of cell divisions in *ABAP1^*OE*^* galls and an increase in cell division rates in *ABAP1/abap1* galls. Also, the repression of *CDT1a* expression in *ABAP1^*OE*^* galls and higher *CDT1a* and *CDT1b* expression in *ABAP1/abap1* galls illustrated their induced gene expression regulation in galls as targets of *ABAP1* transcription repression (Figures 8, 9). Curiously, *E2Fa* expression levels were repressed in both *ABAP1^*OE*^* and *ABAP1/abap1* galls compared to wild-type ones, suggesting that a committed *ABAP1* expression is needed for gall development. In addition, the *SOG1* gene was less expressed in *ABAP1/abap1* galls and did not show difference in *ABAP1^*OE*^* galls compared to wild-type galls, suggesting that *ABAP1* fluctuation in gene expression did not induce check point control activation in galls.

## Discussion

Throughout development, gall cells hyperactivate and regulate their mitotic cell cycle, later undergoing endoreduplication to allow increased ploidy levels (de Almeida-Engler et al., 2015) as described for plant cells (Sugimoto-Shirasu and Roberts, 2003). Aiming to understand DNA replication control through the gall cell cycle, we primarily investigated the expression of representative pre-RC genes that license DNA for replication. The activation of origins of replication results from multisource signaling pathways where positive and negative signals will control the pre-RC machiner (Brasil et al., 2017). Along these lines, we subsequently assessed the function of the ABAP1, a negative G1/S cell cycle regulator, in RKN-induced galls. ABAP1 is a repressor of the DNA replication-licensing machinery for replication, and it regulates the G1 to S phase progression cell rate. Also, ABAP1 acts as a possible sensor of internal and external conditions through its dual role in the regulation of DNA replication and transcription (Masuda et al., 2008). Herein, we show that the right balance of ABAP1 is needed for proper gall development as the knockdown or overexpression of *ABAP1* disturbed gall expansion, as a result affecting nematode development and reproduction. Data on the functional study of ABAP1 in galls as well as transcriptional analysis are summarized in Figure 9.

### Expression of Pre-RC Genes Are Temporally and Spatially Coordinated for Proper Cell Cycle Progression During Gall Development

Licensing DNA for replication involves the activation of origins of replication and the sequential recruitment of proteins to DNA replication origins, establishing the pre-RC, which is the key process in controlling chromosome duplication. Permission to replicate depends on internal and external features so cells can decide when entering the S phase (Brasil et al., 2017). Here, nematodes, by secreting proteins in the root host, hyperactivate the cell cycle, increasing DNA replication levels in a new tumor-like organ, named galls. To find out if similar pre-RC components of uninfected plant cells regulate replication origins in gall cells, the spatial and temporal expression of the pre-RC-members was performed. Assembly and accurate control of pre-RC are crucial prerequisites for cell cycle progression, and we demonstrated here that several elements of the pre-RC are expressed in root-knot nematode-induced galls. We also showed that gall cells expressed several Arabidopsis pre-RC assembly components like *CDC6, MCM5, ORC1, ORC2, ORC3, ORC4, ORC5, ORC6, CDT1a*, and *CDT1b* during the gall mitotic phase (7 DAI) and also during the gall endocycle phase (14 DAI). Gall growth and development is dependent on the mitotic as well as the endoreduplication cycles (de Almeida-Engler et al., 2015). Thus, several of these pre-RC components are likely recruited for the mitotic cycle as well as during the endocycle in developing galls as seen by the presence of transcripts in GCs and NCs including vascular cells. The foremost event in the assembly of the pre-RC is association of ORCs in a complex of six subunits (ORC1–ORC6) to the replication origins containing chromatin marks recognized by ORCs (Bell, 2002; Cunningham and Berger, 2005). All six *ORC*s as well as *CDC6* were shown here to be expressed in galls. Successively, other members of the pre-RC use ORC as a landing platform for CDC6, followed by the recruitment of CDT1s (Bell and Dutta, 2002; Masuda et al., 2004). Besides the ORCs, CDC6 is part of the minimum licensing factor assembling in late G1 during mitosis and the ORCs recruit CDC6 to chromatin further interacting with CDT1s as the cell cycle progresses (Dorn and Cook, 2011; Leonard and Mechali, 2013; MacAlpine and Almouzni, 2013; Pozo and Cook, 2017). In addition, CDC6 and CDT1 proteins act synergistically loading the six DNA helicase MCMs (MCM2–7) opening the replication forks (DePamphilis, 2003; Masai et al., 2005). Also, Bryant (2010) has shown that the *CDC6* gene is expressed during the endoreduplication and, when overexpressed, induced extra endocycles, suggestive of having a similar function in GCs. *MCM5* transcript was detected during gall development with initially higher expression in GCs corroborating with its ubiquitous expression throughout the cell cycle (Springer et al., 2000). MCM5 is a member of the MCM gene family, and MCM5 and MCM7 topologically load onto DNA in plants (Shultz et al., 2009). All through the replicative phase, MCM is shown as the core component of the DNA helicase and, once recruited onto DNA replication origins, leads to replication licensing (Cunningham and Berger, 2005). Thus, the presence of *CDC6*, *MCM5*, and *CDT1a* and *CDT1b* transcripts during the gall mitotic as well as the endocycle phases suggests that nematode-induced galls make use of members of the plant pre-RC machinery in order to activate DNA replication in the host root cell cycle. Possibly, plant–nematode interaction signals driving the cell cycle in galls could engage changes in architecture of pre-RC; thus, other forms of the complex could be altering and hyperactivating the cell cycle in giant-feeding cells. Previous work on plants reported that the cell cycle inhibitor ABAP1 might associate with ORC1a and ORC1b and with pre-RC subunits, or with the fully assembled complex (Masuda et al., 2008). We then investigated ABAP1, a negative cell cycle regulator that controls the assembly of pre-RC in plants (Masuda et al., 2008), to find out if gall ontogeny somewhat underwent this level of cell cycle control.

### ABAP1 Expression Might Control Mitotic Activity in Galls and Its Overexpansion

Promoter activity of *ABAP1* observed during gall development suggested its function as a key regulator of the cell cycle and cell division in galls, as for plants. *ABAP1* expression was formerly observed in the Arabidopsis’ shoot apex, young leaves, flower buds, and dividing root cells, being weakly expressed in lateral roots (Masuda et al., 2008). Higher *ABAP1* promoter activity was reported during early stages of gall development (3 DAI), suggesting that ABAP1 might be regulating entry into S phase, balancing mitosis rates in galls. As galls matured (7–14 DAI), mitotic activity decreased and *ABAP1* expression became weak but still detected. The strong signal observed in flanking roots suggests that nematode infection might control the proliferative state of these bordering root vascular cells. Thus, *ABAP1* expression might prevent galls to expand limiting further mitotic activity. Fainter ABAP1 expression in GCs at later stages of infection (14 DAI) suggests, to a certain level, its involvement during the gall endocycle phase. Endorsement of promoter activity analysis was given by ABAP1 protein localization in galls, where its high expression was obvious in *ABAP1^*OE*^* galls up to 10 DAI (gall endocycle phase) differently from the wild-type where expression decreased. Results of both approaches strongly suggest that *ABAP1* is a candidate to be involved in gall proliferation control. Thus, without a proper cell cycle control, cell cycle hyperactivation occurring in galls might promote uncontrolled growth, leading to a level of root damage that might cause the host death. Thus, gall proliferation must be somewhat controlled by genes regulating the cell cycle such as *ABAP1*. Decreased *ABAP1* expression in maturing galls (10 DAI) corresponds with the entry into the endocycle phase. Overall, ABAP1 expression might likely take part of a highly ordered mechanism of cell cycle control balance preventing gall overexpansion.

### Gall Mitotic Phase Is Prolonged and Endocycle Phase Is Delayed in ABAP1 Knockdown Plants

To assess the role of ABAP1 in galls, as a protein interacting with the pre-RC and directly regulating DNA replication and cell proliferation (Masuda et al., 2008), a mutant T-DNA insertion line having reduced expression levels of *ABAP1* was investigated. Downregulation of *ABAP1* in Arabidopsis did not influence gall induction as gall numbers remained close to the wild-type. However, the impending increased cell cycle activity caused by *ABAP1* knockdown was revealed on the visible changes in gall structure and size and was marked by boosted NC and xylem cell proliferation surrounding GCs. In these GCs, the presence of cell wall stubs indicated an early cytokinesis stimulation. GCs with low *ABAP1* expression levels likewise presented asymmetric and convoluted expansion patterns (14–21 DAI) up to the gall borders but remained apparently smaller compared to the wild-type. Thus, overall, this boosted proliferative state likely caused by the reduced cell cycle inhibition by *ABAP1* led to the increased NCs and xylem division around GCs and their distorted expansion. Nuclear stain of galls with decreased ABAP1 levels shows that as galls matured, the multiple GC nuclei were often clumped and apparently small, likely illustrating the fact that galls remained longer in a proliferative state, delaying progression into the endocycle. Although *ABAP1* knockdown did not affect gall induction, the observed failure in proper gall expansion and reduced GC area resulted in the decreased production of egg masses due to an inhibition on nematode maturation. Thus, these analyses suggest that decreased *ABAP1* expression caused an imbalance of the cell cycle regulation in the feeding site and confirm our previous theory that the mitosis and endocycle offset is needed for a proper gall expansion (de Almeida Engler et al., 2012; de Almeida-Engler et al., 2015; Vieira et al., 2013). The somewhat decreased ploidy levels in GCs of *ABAP1/abap1* line compared to wild-type suggested that activation of nuclei division might delay the endocycle or even that low ABAP1 concentrations might be enough to control the endocycle in galls. Thus, it is reasonable to hypothesize that ABAP1 could participate in a mechanism that control the timing of the switch between the gall mitotic phase to the gall endocycle phase, balancing gall expansion.

### ABAP1 Might Be Participating in a Mechanism That Coordinates the Balance of the Mitotic Cycle and Endocycle During Gall Induction and Expansion

The effect of *ABAP1* upregulation in RKN-induced galls was also accessed here, and its overexpression noticeably exerted a negative function in gall cell proliferation resulting in smaller galls than the ones in wild-type plants. Mitotic activity decreased in NCs and in GCs, possibly by the hindered DNA replication, likewise affecting GC expansion. Furthermore, overexpression of *ABAP1* also hampered gall induction, resulting in lower gall numbers and a decrease in overall nematode reproduction. This inhibitory effect of high ABAP1 levels on gall initiation suggests that an early cell cycle stimulation is primordial for gall induction. Highly vacuolated GCs in *ABAP1^*OE*^* galls and reduced ploidy levels may well illustrate a prompt differentiation and decrease in the high metabolic activity, consequently repressing the mitotic cycle and the endocycle. Also, lower GC ploidy suggests that the ABAP1 might function in mitotic as well as in DNA replication during the endocycle progression in galls. DAPI-stained galls overexpressing *ABAP1* illustrated nuclear but also structural changes in GCs like the presence of cell wall stubs, with aberrant expanded patterns with a poor cytoplasm and grouped nuclei filled with spots of condensed chromatin. These densely stained nuclear spots suggest an increase in heterochromatic regions that might be associated to the decreased endocycle. Components of the pre-RC have been described as players into the heterochromatin assembly, sister chromatin cohesion, chromosome segregation, and cytokinesis in yeast and metazoans, as well as in the epigenetic regulation of transcriptionally repressed regions in yeasts, flies, and mammals (reviewed in Sasaki and Gilbert, 2007; Hemerly et al., 2009). Thus, the interconnected nuclei and compacted chromatin regions remarked upon *ABAP1* overexpression are likely the result of the direct effect of the negative control exerted by ABAP1 on pre-RC in these feeding cells. Overall, the observations suggest that ABAP1 might be participating in a mechanism that coordinates the balance of the mitotic cycle and endocycle during gall induction and expansion, regulating cell proliferation and feeding site homeostasis.

### Genes of Pre-RC Assembly Are Downregulated in *ABAP1^*OE*^* and *ABAP1/abap1* Galls

Expression levels of candidate genes interacting, or directly and indirectly affected by *ABAP1* levels, were examined upon its up- or downregulation, to validate that cell cycle hyperactivation in galls usurps the plant cells’ replication machinery. The expression of the *TCP24* (a class I member of the TCP family) in galls suggests that TCP24 is a potential interactor of ABAP1, and its expression seems not to depend on *ABAP1* expression levels. Alternatively, ABAP1 might regulate cell cycle activity in galls with another partner than TCP24, the ABAP1 interactor in leaves (Masuda et al., 2008). The expression profile of *CDT1a* and *CDT1b* in *ABAP1^*OE*^* and *ABAP1/abap1* galls indicates that an ABAP1–TCP complex might bind *CDT1a* and *CDT1b* promoters in galls, repressing its transcription and regulating cell cycle progression at G1/S in galls and DNA replication.

In galls overexpressing *ABAP1*, the downregulated expression of *CDT1a*, a DNA replication marker, supported the observed phenotype in GCs and their NCs showing inhibited mitotic activity, reinforcing ABAP1 function during the cell cycle in galls. Downregulation of *CDT1a* in *ABAP1^*OE*^* and upregulation of both *CDT1a* and *CDT1b* in *ABAP1* knockdown galls agreed with similar previous observations in leaves of inhibition and stimulation of cell division, respectively (Masuda et al., 2008; Brasil et al., 2017). Thus, low *CDT1a* expression in *ABAP1^*OE*^* galls might be related to the abnormal nuclei phenotype observed in GCs. Also, high *CDT1* levels have been associated with increased endoreduplication (Castellano et al., 2004). Thus, both *CDT1a* and *CDT1b* are likely targets of *ABAP1* in galls and in the control of their expression during DNA replication licensing. Along these lines, *CDT1* might be involved in both DNA replication and also possibly in chromosome segregation; thus, ABAP1 might repress *CDT1a* and *CDT1b* expression, consequently negatively regulating mitotic cell divisions. When downregulated upon high ABAP1 concentration, low *CDT1a* and *CDT1b* levels delay cell cycle progression, driving plants into endoreduplication (Raynaud et al., 2005), similar to what is observed in galls showing increased ploidy levels.

In eukaryotes, members of the pre-RC are regulated by CDK phosphorylation and phosphorylation sites were demonstrated in CDC6 and MCMs from Arabidopsis (Brasil et al., 2017). CDC6 phosphorylation targets the protein for degradation and CDC6 overexpression induces endocycles in Arabidopsis (Bryant and Aves, 2011). Therefore, decreased expression of *CDKA;1* in *ABAP1^*OE*^* galls could contribute to inhibit DNA replication and could participate in the switch to the endocycle phase. In parallel, CDKA;1-CYCB1 complexes are core cell cycle regulators that can be used as markers of active dividing cells (Barrôco et al., 2005). *CYCB1;1* is also a marker of cells at G2 to M phase, being mostly expressed in dividing cells of uninfected roots (Ferreira et al., 1994) and in young galls undergoing the mitotic cycle (Niebel et al., 1996; de Almeida-Engler et al., 1999). Thus, decreased *CDKA;1* expression in *ABAP1^*OE*^* galls could reflect decreased mitotic activity due to *ABAP1* inhibition of DNA replication. No significant changes in *CYCB1;1* level suggests not to be *ABAP1* concentration dependent in *ABAP1^*OE*^* galls. Increased *ABAP1* expression might negatively affect cell cycle progression in galls by targeting genes that regulate phase-to-phase progression, such as *CDT1* and *CDKA;1*, affecting mitotic events and likely contributing to the aberrant nuclei observed in GCs. On the other hand, the upregulation of *CDKA1;1* and *CYCB1;1* seen upon *ABAP1* knockdown is likely associated with the high mitotic activity in galls. No variation in the *PCNA1* mRNA levels in galls suggests that its expression is independent of *ABAP1* levels and that minimum concentration might be enough to hold polymerase activity. Also, in plants, *PCNA1* and its paralogous *PCNA2* are highly conserved genes, with similar sequences, structures, and pattern of protein–protein interaction (Qian et al., 2019), suggesting a redundant function that might overcome some imbalances in their expression as for in galls. PCNA1 is an auxiliary protein of DNA polymerase involved in the control of eukaryotic DNA replication and is the major coordinator of DNA repair at replication forks (Kosugi and Ohashi, 2002; Stevens et al., 2002). Stress-induced effects on the cell cycle has been shown to occur in galls by induction of check point control, likely as a DNA damage response (Cabral et al., 2020). The transcription factor *SOG1* was not activated in galls of both *ABAP1^*OE*^* and *ABAP1/abap1* lines, and its expression was significantly decreased upon *ABAP1* downregulation. Therefore, DNA repair mechanism seems not to be activated upon changed *ABAP1* levels. No increase in *SOG1* expression in galls could also be associated with the reduced stress caused in the host root seen by the smaller galls in both *ABAP1^*OE*^* and *ABAP1/abap1* lines. Finally, we monitored the expression of *E2Fa*, a member of E2F transcription factors involved in the mitotic as well as the endocycle in plants. Transcription of *E2Fa* promotes the expression of pre-RC genes that bind to DNA replication origins at G1 phase (Castellano et al., 2001, 2004; Diaz-Trivino et al., 2005). *E2Fa* levels in galls in both *ABAP1^*OE*^* and *ABAP1/abap1* lines were reduced likely due to the decreased cell cycle activity observed in both transgenic lines.

## Concluding Remarks

Our results provide strong evidences that galls induced by parasitic root-knot nematodes usurp the pre-RC of plant hosts in order to hyperactivate their cell cycle (Figure 9). Even when mitotic activity ceases in galls, transcription of pre-RC components here studied remained during DNA replication through the endocycle, suggesting the use of a common regulation of the DNA replication machinery in both phases. Divergences between pre-RC control in galls compared to uninfected roots might occur, given that variations in expression levels were observed, likely reflecting slight differences in strategies of cell cycle regulation. Our functional data indicate that in RKN-induced galls, *ABAP1* can play a novel role during the G1/S phase progression on the course of the endocycle. *ABAP1* upregulation arrested gall development and knockdown perturbed gall homeostasis, suggesting that the accurate cell cycle regulation in GCs is of utmost importance to avoid the accumulation of mitotic defects leading to aberrant nuclear phenotypes. ABAP1 might also be mainly necessary to slow down gall expansion and development, controlling proliferative cell divisions leading to size control during this unique nematode-induced organ growth. Thus, we hypothesize that ABAP1 operates as a negative regulator of the S phase in the mitotic cell cycle and endocycle in galls, possibly participating on NFS induction and development. Most likely, ABAP1 has TCP24 as a partner negatively regulating *CDT1a* expression and then disturbing the pre-RC assembly impeding proper DNA replication and gall mitotic activity. In addition, signaling controls coming from nematode infection might be involved in regulating steps during DNA replication. Finally, understanding the function of cell cycle control genes directing gall homeostasis may well be useful to generate tools enhancing plant resistance to cell cycle-dependent pathogens like nematodes.

## Data Availability Statement

The original contributions presented in the study are included in the article/Supplementary Material, further inquiries can be directed to the corresponding author/s.

## Author Contributions

JA, DC, and HF conceived and designed the experiments. DC, JA, HF, IL-T, BM, KS, and LO performed the experiments. JA, DC, HF, and AH analyzed the data. AH and MG contributed reagents, materials, and analysis tools. JA, DC, and BM wrote the manuscript. JA and AH amended the manuscript. All authors contributed to the article and approved the submitted version.

## Conflict of Interest

The authors declare that the research was conducted in the absence of any commercial or financial relationships that could be construed as a potential conflict of interest.

## Supplementary Material

Supplementary Figure 1 Spatial localization of *CDT1a* and *CDT1b* transcripts by non-radioactive *in situ* hybridization in *Meloidogyne incognita*-induced galls of Arabidopsis. Bright-field images of mRNA *in situ* hybridization in nematode feeding sites (orange dotted lines) 7 and 14 DAI. Sections were hybridized with DIG-labeled antisense (AS) probes. *CDT1a* and *CDT1b* hybridization signal is shown as purple color under bright-field optics. UR, uninfected root; DAI, days after inoculation; VT, vascular tissue; DAI, days after inoculation; n, nematodes. Bars = 50 μm.

Supplementary Table 1 List of primer sequences used for qRT-PCRPCR analysis.

Supplementary Movie 1 Confocal projections of serial optical sections of a gall 30 DAI induced by *M. incognita* in Arabidopsis- Wild-type.

Supplementary Movie 2 Confocal projections of serial optical sections of a gall 30 DAI induced by *M. incognita* in Arabidopsis-*ABAP1/abap1.*

Supplementary Movie 3 Confocal projections of serial optical sections of a gall 30 DAI induced by *M. incognita* in Arabidopsis-Wild-type.

Supplementary Movie 4 Confocal projections of serial optical sections of a gall 30 DAI induced by *M. incognita* in Arabidopsis-*ABAP1^*O**E*^.*
