# Supplementary figures and images for: The Armadillo BTB Protein ABAP1 Is a Crucial Player in DNA Replication and Transcription of Nematode-Induced Galls
Source: Front Plant Sci. 2021 Apr 30;12:636663. doi: 10.3389/fpls.2021.636663 (PMC8121025; doi:10.3389/fpls.2021.636663)

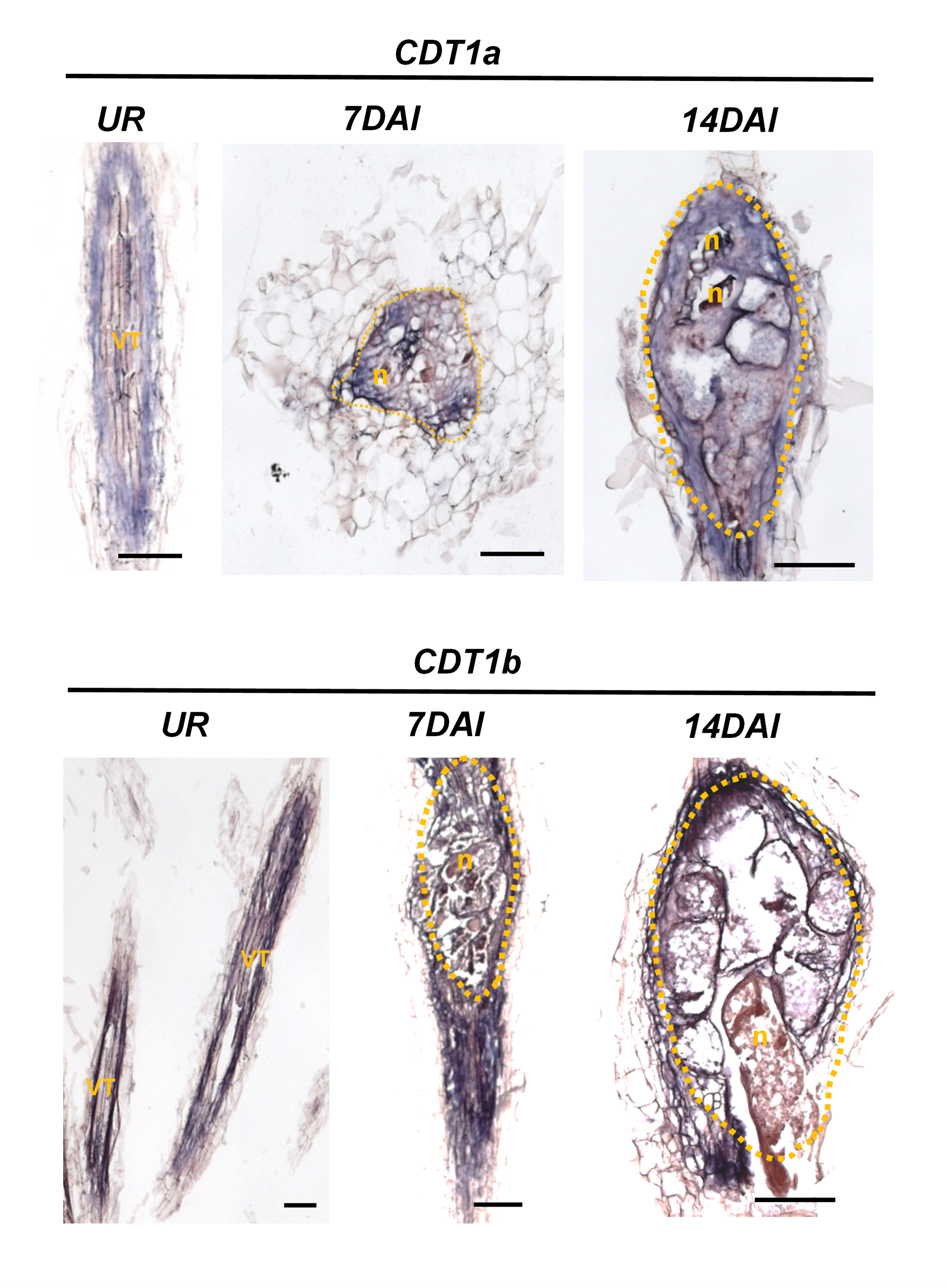

Supplement: Supplementary Figure 1 — Spatial localization of CDT1a and CDT1b transcripts by non-radioactive in situ hybridization in Meloidogyne incognita-induced galls of Arabidopsis. Bright-field images of mRNA in situ hybridization in nematode feeding sites (orange dotted lines) 7 and 14 DAI. Sections were hybridized with DIG-labeled antisense (AS) probes. CDT1a and CDT1b hybridization signal is shown as purple color under bright-field optics. UR, uninfected root; DAI, days after inoculation; VT, vascular tissue; DAI, days after inoculation; n, nematodes. Bars = 50 μm. [file Image_1.TIF]
